# Supplementary material for: Neurotransmission Sex Dichotomy in the Rat Hypothalamic Paraventricular Nucleus in Healthy and Infantile Spasm Model
Source: Curr Issues Mol Biol. 2025 May 21;47(5):380. doi: 10.3390/cimb47050380 (PMC12110120; doi:10.3390/cimb47050380)
Supplement: Supplementary file 1 [file cimb-47-00380-s001.zip › cimb-3578405-supplementary.pdf]

Supplementary Material

	**Top 5 expressed neurotransmission genes**		**M/F**		
**Gene**	**Description**	**PATH**	**SNS**	**BNS**	**BYS**
Caly	**calcyon neuron-specific vesicular protein**	4	-1.55	-1.03	-1.23
Gabarapl1	GABA(A) receptor-associated protein like 1	2	1.49	1.03	-1.61
Ap2m1	adaptor-related protein complex 2, mu 1 subunit	0	-1.31	-1.02	-1.37
Calm2	calmodulin 2	4	1.20	-1.07	-1.20
Hap1	huntingtin-associated protein 1	2	-1.16	-1.15	-1.09
Caly	**calcyon neuron-specific vesicular protein**	4	-1.55	-1.03	-1.23
Ap2m1	adaptor-related protein complex 2, mu 1 subunit	0	-1.31	-1.02	-1.37
Mapk3	mitogen activated protein kinase 3	135	-1.44	-1.06	-1.01
Gnai2	guanine nucleotide binding protein (G protein), alpha inhibiting activity polypeptide 2	12345	-1.31	1.01	-1.30
Hap1	huntingtin-associated protein 1	2	-1.16	-1.15	-1.09
Caly	**calcyon neuron-specific vesicular protein**	4	-1.55	-1.03	-1.23
Gabarapl1	GABA(A) receptor-associated protein like 1	2	1.49	1.03	-1.61
Ap2m1	adaptor-related protein complex 2, mu 1 subunit	0	-1.31	-1.02	-1.37
Calm2	calmodulin 2	4	1.20	-1.07	-1.20
Gnai2	guanine nucleotide binding protein (G protein), alpha inhibiting activity polypeptide 2	12345	-1.31	1.01	-1.30
Caly	**calcyon neuron-specific vesicular protein**	4	-1.55	-1.03	-1.23
Ap2m1	adaptor-related protein complex 2, mu 1 subunit	0	-1.31	-1.02	-1.37
Gabarapl1	GABA(A) receptor-associated protein like 1	2	1.49	1.03	-1.61
Calm2	calmodulin 2	4	1.20	-1.07	-1.20
Hap1	huntingtin-associated protein 1	2	-1.16	-1.15	-1.09
Caly	calcyon neuron-specific vesicular protein	4	-1.55	-1.03	-1.23
Ap2m1	adaptor-related protein complex 2, mu 1 subunit	0	-1.31	-1.02	-1.37
Gabarapl1	GABA(A) receptor-associated protein like 1	2	1.49	1.03	-1.61
Mapk3	mitogen activated protein kinase 3	135	-1.44	-1.06	-1.01
Hap1	huntingtin-associated protein 1	2	-1.16	-1.15	-1.09
Caly	**calcyon neuron-specific vesicular protein**	4	-1.55	-1.03	-1.23
Gabarapl1	GABA(A) receptor-associated protein like 1	2	1.49	1.03	-1.61
Ap2m1	adaptor-related protein complex 2, mu 1 subunit	0	-1.31	-1.02	-1.37
Gnai2	guanine nucleotide binding protein (G protein), alpha inhibiting activity polypeptide 2	12345	-1.31	1.01	-1.30
Hap1	huntingtin-associated protein 1	2	-1.16	-1.15	-1.09
Cst3	cystatin C		-1.30	-1.09	-1.20
Rpl41	ribosomal protein L41		-1.53	1.00	-1.39

Table S1: Expression ratios (negative for down-regulation) of the top 5 expressed genes between the two sexes in the three conditions. Path = 0 (SVC), 1 (GLU), 2 (GAB), 3 (CHO), 4 (DOP), 5 (SER).

	Top 5 most controlled neurotransmission genes		M-RCS/F-RCS		
Gene	Description	PATH	SNS	BNS	BYS
Pik3r2	**phosphoinositide-3-kinase, regulatory subunit 2 (beta)**	3	6.44	1.22	1.07
Atp6v0b	ATPase, H+ transporting, lysosomal V0 subunit B	0	2.61	-1.64	2.36
Calml4	calmodulin-like 4	4	6.56	-1.50	2.57
Ppp1ca	protein phosphatase 1, catalytic subunit, alpha isozyme	4	1.95	-2.72	-1.15
Atp6v1g2	ATPase, H+ transporting, lysosomal V1 subunit G2	0	3.71	-2.27	-1.65
Abat	**4-aminobutyrate aminotransferase**	2	-9.24	-1.22	2.46
Gabbr1	gamma-aminobutyric acid (GABA) B receptor 1	2	-8.77	1.48	-1.40
Slc6a7	solute carrier family 6 (neurotransmitter transporter), member 7	0	-3.35	1.05	-1.54
Chrnb4	cholinergic receptor, nicotinic, beta 4 (neuronal)	3	-3.68	-1.42	1.22
Gnal	GTP-binding protein Golf alpha subunit	4	-3.57	-1.63	1.02
Pik3r5	**phosphoinositide-3-kinase, regulatory subunit 5**	3	-2.28	12.21	1.06
Atp2a2	ATPase, Ca++ transporting, cardiac muscle, slow twitch 2	0	2.29	1.57	-1.10
Ppp2r5e	protein phosphatase 2, regulatory subunit B', epsilon isoform	4	2.29	2.28	3.04
Ppp2r3c	protein phosphatase 2, regulatory subunit B'', gamma	4	4.05	3.35	-1.40
Slc38a5	solute carrier family 38, member 5	2	4.10	6.44	-1.35
Grm8	**glutamate receptor, metabotropic 8**	1	-1.16	-7.70	2.16
Cpne3	copine III	0	2.31	-3.26	1.72
Atp6v1h	ATPase, H+ transporting, lysosomal V1 subunit H	0	2.06	-1.67	-1.05
Gls	glutaminase (Gls), nuclear gene encoding mitochondrial protein	12	2.60	-2.17	1.42
Gabarapl2	GABA(A) receptor-associated protein like 2	2	4.17	-2.77	-1.26
Th	**tyrosine hydroxylase**	4	-3.53	-1.28	14.58
Maoa	monoamine oxidase A	45	-1.49	-1.32	8.21
Mapk10	mitogen activated protein kinase 10	4	6.98	1.27	3.78
Scn1a	sodium channel, voltage-gated, type I, alpha	4	-2.46	2.57	3.97
Gabrg1	gamma-aminobutyric acid (GABA) A receptor, gamma 1	2	-1.47	-3.30	8.54
Trpc1	**transient receptor potential cation channel, subfamily C, member 1**	15	3.09	1.27	-8.01
Gng10	guanine nucleotide binding protein (G protein), gamma 10	12345	1.42	-1.23	-6.43
Gng8	guanine nucleotide binding protein (G protein), gamma 8	1245	-1.50	1.46	-4.52
Raf1	v-raf-leukemia viral oncogene 1	5	1.05	4.99	-2.88
Atp6v0a1	ATPase, H+ transporting, lysosomal V0 subunit A1	0	1.39	-3.87	-1.28
Erg28	**ergosterol biosynthesis 28 homolog**		18.61	-1.62	-1.78
Erap1	**endoplasmic reticulum aminopeptidase 1**		-23.73	-1.52	-1.78
Tmem238	**transmembrane protein 238**		1.03	23.60	1.78
Cul7	**cullin-7**		1.63	-11.19	-1.66
Oxsr1	**oxidative-stress responsive 1**		-2.71	-3.74	21.86
Tsc2	**tuberous sclerosis 2**		-1.48	1.40	-34.07

Table S2: Ratios of the Relative Strength Controls (negative for down-regulation) of the most 5 controlled genes between the two sexes in the three conditions. Path = 0 (SVC), 1 (GLU), 2 (GAB), 3 (CHO), 4 (DOP), 5 (SER).

	Top 5 least controlled neurotransmission genes		M-RCS/F-RCS		
**Gene**	**Description**	**PATH**	**SNS**	**BNS**	**BYS**
Calm1	**calmodulin 1**	4	-1.71	-1.24	-2.15
Alox15	arachidonate 15-lipoxygenase	5	-4.01	1.78	1.21
Th	tyrosine hydroxylase	4	-3.53	-1.28	14.58
Plcb1	phospholipase C, beta 1 (phosphoinositide-specific)	1345	-2.76	-1.26	1.43
Clock	clock circadian regulator	4	-1.30	-1.64	1.03
Mapk10	**mitogen activated protein kinase 10**	4	6.98	1.27	3.78
Gna11	guanine nucleotide binding protein, alpha 11	3	2.32	-1.80	1.19
Gabarapl1	GABA(A) receptor-associated protein like 1	2	2.02	1.01	-1.29
Rims1	regulating synaptic membrane exocytosis 1	0	1.43	-1.25	1.23
Map2k1	mitogen activated protein kinase kinase 1	35	2.18	-1.79	1.69
Drd2	**dopamine receptor D2**	4	1.08	-1.81	1.25
Slc6a12	solute carrier family 6 (neurotransmitter transporter), member 12	02	1.03	-2.08	1.21
Clock	clock circadian regulator	4	-1.30	-1.64	1.03
Alox12b	arachidonate 12-lipoxygenase, 12R type	5	-1.86	-1.64	1.20
Gnb3	guanine nucleotide binding protein (G protein), beta polypeptide 3	12345	1.05	-1.61	1.18
Creb3	**cAMP responsive element binding protein 3**	34	1.05	9.98	1.15
Gng5	guanine nucleotide binding protein (G protein), gamma 5	12345	1.19	6.72	-1.18
Camk2b	calcium/calmodulin-dependent protein kinase II beta	34	3.94	2.93	1.36
Akt1	v-akt murine thymoma viral oncogene homolog 1	34	1.89	3.76	2.23
Slc1a3	solute carrier family 1 (glial high affinity glutamate transporter), member 3	01	-1.38	3.01	-1.51
Clock	**clock circadian regulator**	4	-1.30	-1.64	1.03
Gria2	glutamate receptor, ionotropic, AMPA 2	14	2.75	-2.44	-2.50
Htr7	5-hydroxytryptamine (serotonin) receptor 7, adenylate cyclase-coupled	5	-1.11	-1.42	1.08
Unc13b	unc-13 homolog B (C. elegans)	0	-1.78	-1.50	1.13
Akt2	v-akt murine thymoma viral oncogene homolog 2	34	1.10	-1.80	1.06
Grin1	**glutamate receptor, ionotropic, N-methyl D-aspartate 1**	1	-1.26	-1.38	1.39
Gnaq	guanine nucleotide binding protein (G protein), q polypeptide	1345	-1.78	-1.63	1.33
Chrna7	cholinergic receptor, nicotinic, alpha 7 (neuronal)	3	-1.38	-1.57	1.23
Cyp2c11	cytochrome P450, subfamily 2, polypeptide 11	5	1.31	-1.23	1.83
Gnb3	guanine nucleotide binding protein (G protein), beta polypeptide 3	12345	1.05	-1.61	1.18
C11h21orf91	similar to human chromosome 21 open reading frame 91		-3.53	-1.90	1.22
Snx5	sorting nexin 5		14.61	2.01	-1.46
Pddc1	Parkinson disease 7 domain containing 1		-2.14	-13.36	1.53
Mobp	myelin-associated oligodendrocyte basic protein		-1.01	1.66	-1.31
Pfkl	phosphofructokinase, liver		4.65	4.56	-4.77
Ptgds	prostaglandin D2 synthase (brain)		-2.64	-2.22	3.52

Table S3: Ratios of the Relative Strength Controls (negative for down-regulation) of the laest controlled genes between the two sexes in the three conditions. Path = 0 (SVC), 1 (GLU), 2 (GAB), 3 (CHO), 4 (DOP), 5 (SER).
